# The Importance of Land Use Definition in Human Health Risk Assessment Related to Lead in Soils

**DOI:** 10.1155/2019/7973890

**Published:** 2019-10-28

**Authors:** Ricardo Urrutia-Goyes, Nancy Ornelas-Soto

**Affiliations:** ^1^Departamento de Ciencias de la Energía y Mecánica, Universidad de las Fuerzas Armadas ESPE, Av. Gral. Rumiñahui s/n, P.O. Box 171-5-231B, Sangolqui 171103, Ecuador; ^2^Laboratorio de Nanotecnología Ambiental, Tecnologico de Monterrey, Escuela de Ingeniería y Ciencias, Ave. Eugenio Garza Sada 2501, Monterrey, NL 64849, Mexico

## Abstract

In many countries, soil contamination and lead exposure is a persistent human and environmental health issue, while in others, it is an emerging concern. Defining the extent of lead contamination and assessing human health risk allow for efficient prevention agendas. The different types of land uses delimit the exposure frequency and hence can influence the evaluation of possible threats. In this study, human health risk assessment is performed under different land use scenarios, after determining the concentration of lead in topsoil of a rehabilitated space. An analytical hybrid method was used to determine the concentrations of the heavy metal. Human health risk indicators, hazard quotient and cancer risk, were subsequently calculated and compared under such scenarios of varying population exposure by land use. Results indicate that an increasing exposure can set health risk indicators above the tolerable levels. Correctly defining the exposure frequency by land use is very important to determine the actual risk levels of a site. Local regulators should take this information into account before designing prevention plans, especially in localities where migration and urbanization are major development factors and since the land use of a public place could change over time and alter the exposure frequency to soil.

## 1. Introduction

In a never-ending urbanizing and industrializing world, exposure to Pb in soils is an overlooked global concern. In developed countries, there still are several sites where Pb contamination has not been remediated, while in developing countries, Pb contamination is only starting to be reckoned. Migration phenomena between the two types of countries can also contribute to increasing exposure to contaminants since exposure often coincides with development conditions such as access to redeveloped spaces turned into urbanized areas. Since Pb not only causes noncarcinogenic effects on the population but is also considered a class B2 carcinogen by WHO and USEPA [1, 2], its monitoring and evaluation should be continuous in order to avoid repercussions for future generations.

Soils can have high Pb content due to anthropogenic or natural activities. Soils contaminated with Pb can pose risks, since population exposed to the metal by ingestion or inhalation may show health complications such as neurological, renal, and cardiovascular [3]. In order to avoid such exposure, the first step is to carry out an analytical characterization of soils with the goal of detecting high Pb concentrations. USEPA sampling and analytical methods [4–6] are used for most soil characterizations, where the principal techniques recommended for the determination of inorganic analytes include atomic absorption (AA), inductively coupled plasma mass spectrometry (ICP-MS), and X-ray fluorescence (XRF) [7, 8]. Nonetheless, methods that involve combined analytical techniques have also been successfully used lately in environmental analysis [9, 10].

The second step to minimize the exposure related to Pb pollution is to perform risk assessment. Human health risk assessment seeks to determine carcinogenic risks and noncarcinogenic hazards caused when the population is exposed to toxic chemicals present in soil or other media. One of the parameters calculated in the assessment is the chronic daily intake value (CDI). CDI values represent the amount of chemical substance that a person receives over a period of time. CDI values, and hence risk indicators, depend on a factor called exposure frequency (EF), which is the number of days per year that a person is assumed to be in contact with a toxic chemical substance. EF values vary according to the land use of the study area. For instance, in a scenario of residential land use, residents are in repeated contact with the contaminated soil for ∼350 days per year; in a scenario of industrial land use, workers are exposed for ∼250 days based on potential equipment use; and in a scenario of recreational land use, adults and children spend only ∼40 days in contact with the media since such activities are occasional. This way, different types of land use determine different exposure frequencies and influence the evaluation of risks. Finally, the third step to minimize metal exposure is to socialize results with local regulators to raise awareness and propose possible guidelines.

In this study, human health and ecological risk assessment is performed under different land use scenarios after characterizing Pb across a public area. The main goal is to determine the influence of exposure to Pb in the evaluation of risks since land use scenarios are a determining factor in the calculation of chronic daily intake values. Local and global regulators could benefit from these results since the correct determination of land use and exposure frequency values can improve any future monitoring and prevention plans.

## 2. Materials and Methods

### 2.1. Sampling and Analytical Procedures

**Figure 1:**
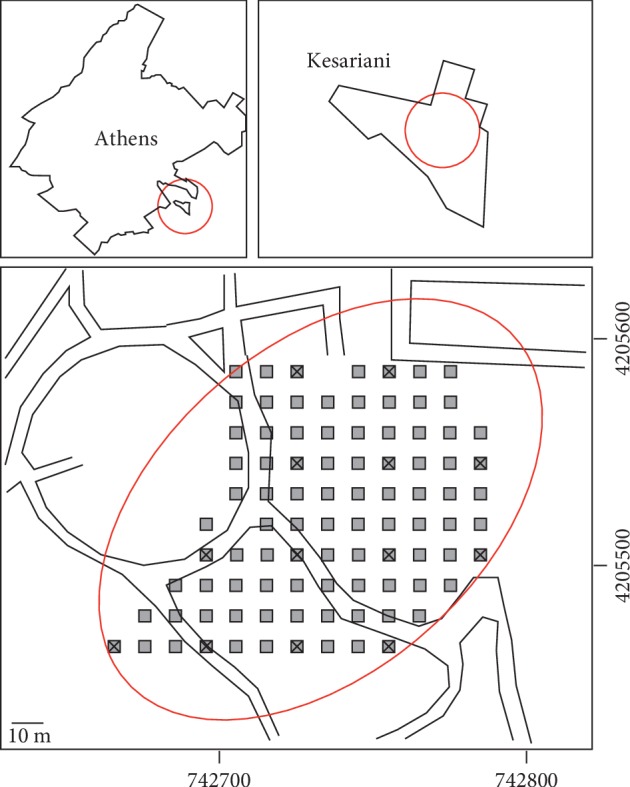
Sampling locations for Pb exposure analysis. Gray squares represent samples analyzed by using XRF only. Crossed grayed squares represent samples analyzed by XRF and ICP-MS.

A public place in Athens, Greece, was referenced by using a GPS and long measuring tape according to Figure 1. A total of 91 superficial soil samples were collected by removing 400 g of topsoil (0–20 cm) with a spatula. Any vegetation residues or gravel were removed from the samples before storing them in commercial polypropylene bags. After transportation to the laboratory, samples were dried for 24 h at 60° and disaggregated in a mortar. Before analytical procedures, samples were quartered and sieved to <250 *μ*m particle size in order to improve homogeneity of the subsamples. Loss of sample soil due to this treatment was insignificant [4, 11, 12]. Samples were collected during the first semester of 2016. The study area is a redeveloped park where people gather for leisure since the 2000s and is surrounded by urbanized streets where commercial and residential infrastructure can be found. The area was a recreational shooting range in the past. It has an area of ∼70 ha and is covered by simple vegetation and coniferous trees.

Analytical techniques used to obtain Pb concentration in soil samples were ICP-MS and XRF. The samples were analyzed both by XRF directly (91 samples) and ICP-MS after total acid digestion (13 samples), in order to obtain 91 corrected XRF measurements that match ICP-MS accuracy following a hybrid methodology [9, 10]. Details on the hybrid methodology can be found in the previous publications [10, 13]. Briefly, to obtain XRF measurements, samples were kept inside their plastic bags and homogenized. The nose of the device was placed on top of the sample keeping a layer of soil of at least 3 cm below. The measurements were taken for 90 s. As part of the quality control process, the following certified reference materials were used: NIST 2710a, NIST 2711a, and CRM 023 [7]. On the other hand, to obtain ICP-MS measurements, samples were sieved to <250 *μ*m particle size, and the resulting fraction was dissolved with nitric acid and subsequently diluted into a 100 mL volumetric flask with deionized water. The quality control process included certified reference materials, blanks, and duplicates [7, 14, 15]. Finally, the data obtained by using XRF were fitted to match the corresponding measurements obtained by using ICP-MS. The linear regression (trend equation) calculated between these data points was applied to the rest of the XRF data points to match accuracies with constant correlation (*R*^2^ > .090) [9, 10].

### 2.2. Reagents and Instrumentation

Reagent-grade chemicals and deionized water were used in the experimentation. Specific acids and stock solutions were obtained from Sigma-Aldrich (St. Louis, MO, USA). Certified reference materials were obtained from NIST (Gaithersburg, MD, USA). Instrumental analysis was carried out by XRF using an Olympus (Newton, MA, USA) Delta Premium 6000 device and ICP-MS using a Thermo Fisher (Waltham, MA, USA) X Series 2 instrument.

### 2.3. Human Health and Ecological Risk Assessment

Health risk indicators, hazard quotient (HQ) and cancer risk (CR), were calculated for Pb [3, 16–21]. Acceptable levels for total HQ values are smaller than one while tolerable levels for total CR values are in the range of 10^−6^–10^−4^ [16–18, 20, 21]. Calculations were based on the following parameters:

1. Chronic daily intake (CDI) value depends on the concentration of the metal across the site. CDI values represent in this case the amount of Pb absorbed by a person over the studied period. Each route of exposure defines its respective CDI value (CDI_ing_, CDI_inh_, and CDI_dermal_) and was calculated according to equations (1)–(3). Specific additional parameters can be seen in Table 1.
2. Reference dose (RfD) values are 3.5 × 10^−3^ mg/kg/day for ingestion/inhalation and 5.25 × 10^−4^ mg/kg/day for dermal contact. RfD represents the maximum daily Pb exposure that would not yield effects on the population. HQ values were calculated according to equation (4). The total HQ value is obtained by adding the correspondent singular values regarding ingestion, inhalation, and dermal contact since effects can be considered accumulative [16].
3. A slope factor (SF) value of 8.5 × 10^−3^ mg/kg/day allows the calculation of CR according to equation (5). SF represents the incremental chance of a person to develop Pb-related cancer over a lifetime under the mentioned exposure.

The ecological risk indicator, Potential Ecological Risk Index (RI), was calculated for the study area in the total absence or presence of Pb following equation (6). RI allows an evaluation of potential adverse ecological effect of metals in soil based on toxic response factors (*T*_r_^i^) of 10, 2, 30, 5, 1, 5, and 5 for As, Cr, Cd, Cu, Zn, Pb, and Ni [22, 23]. Such metals were analyzed for every sampling point according to the same procedure followed for Pb. *B*_*n*_ values were obtained from Rudnick and Gao [24]. Calculated RI values greater than 150 define a moderate risk, greater than 300 a considerable risk, and greater than 600 a high ecological risk.

(1) $$\begin{array}{c} {\text{CDI}}_{\text{ing}}=C_{\exp}\times \frac{R_{\text{ing}}\times \text{EF}\times \text{ED}}{\text{BW}\times \text{AT}}\times 10^{-6}, \end{array}$$

(2) $$\begin{array}{c} {\text{CDI}}_{\text{inh}}=C_{\exp}\times \frac{R_{\text{inh}}\times \text{EF}\times \text{ED}}{\text{PEF}\times \text{BW}\times \text{AT}}, \end{array}$$

(3) $$\begin{array}{c} {\text{CDI}}_{\text{dermal}}=C_{\exp}\times \frac{\text{SA}\times \text{SAF}\times \text{ABS}\times \text{EF}\times \text{ED}}{\text{BW}\times \text{AT}}\times 10^{-6}, \end{array}$$

(4) $$\begin{array}{c} \text{HQ}=\frac{\text{CDI}}{\text{RfD}}, \end{array}$$

(5) $$\begin{array}{c} \text{CR}=\text{CDI}\times \text{SF,} \end{array}$$

(6) $$\begin{array}{c} \text{RI}=\overset{7}{\underset{i=1}{\sum }}T_{r}^{i}\left(\frac{C_{n}}{B_{n}}\right). \end{array}$$

### 2.4. Lead Exposure Influence

The exposure to Pb in soil from public places occurs when soil particles come in contact with the human body (mouth, nose, or skin). Additionally, the abovementioned indicators of human health risk assessment depend on the EF (exposure frequency) parameter defined in Table 1. However, the definition of exposure to Pb in the study area can be complex since the park's focus is recreational but commercial and residential buildings also surround the whole area. Initially, EF parameter was set to 40 for recreational land use, but people can be exposed to such soils for more than 40 days every year. This way, additional HQ and CR calculations were performed with different EF values (20, 40, 100, 150, 180, 250, and 350) in order to analyze the influence that varying exposure frequencies to Pb would cause. Plots were prepared to show the variation of both indicators, and maps were devised to show the variation in spatial risk for adults and children due to Pb contamination with increasing exposure.

### 2.5. Statistical Analysis

Descriptive statistics and regression analysis calculated for Pb included median, mean, and standard deviation. Statistical analysis was performed by using software packages XLSTAT® (Addinsoft, Paris, France 2017) and Minitab® (State College, PA, USA). Spatial representation of human health risk assessment was performed by using the SADA software of the University of Tennessee Knoxville (Knoxville, TN, USA).

## 3. Results and Discussion

Accuracy and precision analyses showed a range of RSD (relative standard deviation) values from 1 to 2%, and mean recoveries calculated were 108%, for XRF. Calculated RPD (relative percentage difference) and mean recoveries for ICP-MS were 8 and 103%, respectively. Pb concentrations in topsoil show a range from 43 to 86,000 mg/kg. Such a wide range of values can be attributed to an extremely heterogeneous metal distribution, which in turn coincides with the abovementioned method of Pb deposition (shooting range). The mean concentration is 7,160 mg/kg, and the standard deviation is 12,689 mg/kg, which confirms a high heterogeneity in the study area [25, 26].

### 3.1. Human Health and Ecological Risk Assessment

Descriptive calculated parameters resulted as follows.

The total CDI value for adults was 3.94 × 10^−3^ based on a Pb concentration of 25,067 mg/kg (95% UCL) and as a result of adding partial CDI values 3.92 × 10^−3^, 1.15 × 10^−6^, and 1.57 × 10^−5^ for ingestion, inhalation and dermal contact, respectively. Likewise, total CDI was 3.67 × 10^−2^ for children. It can be noted that the main exposure contributions for Pb follow the order ingestion > dermal contact > inhalation. It can be suggested that at least some policies should be put in place to protect the people who gather around the study area. Children, specially, are prone to play in the dirt and hence would have a greater chance of Pb intake due to high concentrations of the heavy metal. The CDI value for children is almost ten times bigger than the one for adults and hence the calculated risk will be similarly greater.

The total HQ value for adults was 1.15 × 10^0^, while the total HQ value for children was 1.07 × 10^+1^. HQ values for ingestion, inhalation, and dermal contact in adults were 1.12 × 10^0^, 3.30 × 10^−4^, and 2.98 × 10^−2^, respectively. The tendency ingestion > dermal contact > inhalation remains. Noncarcinogenic hazard related to Pb contamination in the study area is ten times bigger for children than for adults and is greater than one for both adults and children, meaning that there is a real threat for the residents of the area. Such HQ values are much greater than others reported in parks and playgrounds from Spain, Turkey, Portugal, Montenegro, and China [17, 20, 27–29]. However, these additional reports use different exposure frequency values and hence comparisons are referential only. There appears to be some influence of the exposure frequency in the resulting noncarcinogenic hazard evaluated.

The CR value for adults was 1.14 × 10^−5^, while the CR value for children was 2.67 × 10^−5^. Carcinogenic risk related to Pb contamination in the study area is two times bigger for children than for adults. In both cases, CR values fall inside the tolerable range of 10^−6^–10^−4^ but are greater than others reported in parks and playgrounds from China [20] with different exposure frequencies. Once again, the EF value seems to influence the results for carcinogenic risk.

On the other hand, the mean RI value for the study area depended on the mean concentration of As (6.2 mg/kg), Cr (177.9 mg/kg), Cd (not detected), Cu (32.6 mg/kg), Zn (75.0 mg/kg), Pb (7,160 mg/kg), and Ni (92.0 mg/kg) detected in the soil samples. The total RI value calculated was 2,022, which is far above 600, the limit for high ecological risk.

### 3.2. Lead Exposure Analysis

**Figure 2:**
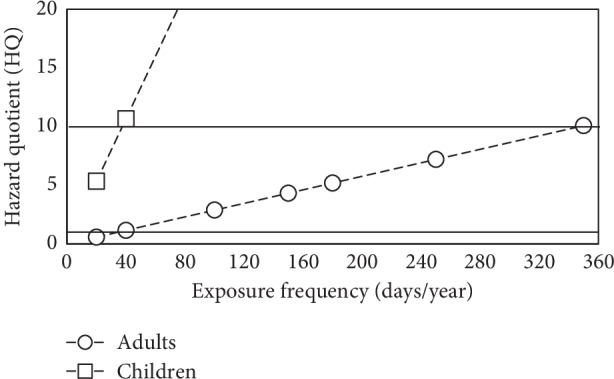
Noncarcinogenic Pb-related hazard values for adults and children based on different exposure frequencies.

Figure 2 shows how the calculated hazard quotient (HQ) values increase according to different exposure frequency (EF) values. Reference lines for HQ = 1 and HQ = 10 can be seen for comparison purposes since HQ values greater than one yield risk scenarios. It can be seen that Pb in the study area does not pose a health risk for adults only if exposure frequencies smaller than 40 days/year are considered. This can be the case for scenarios of excavation land use (EF = 20). However, increasing EF values for different land uses yield HQ values for adults of 1.15 (recreational, EF = 40), 7.19 (industrial, EF = 250), and 10.01 (residential or agricultural, EF = 350), which pose a significant health risk. In the case of children, it can be noted that even the smallest EF value, for excavation land use, yields HQ values greater than one and increases linearly to very noteworthy risk values. Properly defining EF values according to land use scenarios is very important in order to accurately perform risk assessment by local authorities for monitoring or possible posterior treatment.

**Figure 3:**
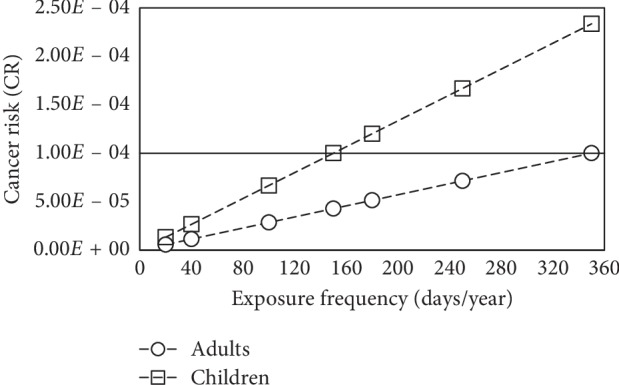
Carcinogenic Pb-related risk values for adults and children based on different exposure frequencies.

In the same way, Figure 3 shows the calculated cancer risk (CR) values. When exposure frequency (EF) values increase, the indicator also increases. A reference line of 1.00 × 10^−4^ can be seen since the tolerable zone is in the range of 10^−6^-10^−4^. As can be noted, the study area sits in the tolerable cancer risk zone for adults. However, an exposure frequency greater than 350, for residential or agricultural land use, would be in the limit between the tolerable area and the cancer risk area. In the case of children, Pb-related cancer risk sits in the tolerable zone for exposure frequencies smaller than 160, which includes excavation and recreational land use scenarios. On the other hand, the CR value becomes present and a real threat for exposure frequencies greater than 160, which includes industrial, residential, and agricultural land use scenarios. It would be important for local authorities, to define the precise exposure frequency that adults and children of the area have with respect to Pb, in order to determine whether a possible cancer risk is feasible.

Once it has been established that human health and ecological risk assessment depend on the exposure frequency chosen for a given study area, it is relevant to analyze such variations on a spatial scale in addition to a general scale. In the previous paragraphs, HQ, CR, and RI values were calculated using a concentration value for Pb in soil that represents the 95% upper confidence limit for measured values. This Pb concentration value was used to generate one indicator that covers the whole site. However, it is understood that every location on the area has a different Pb concentrations and hence should yield different human health and ecological indicators. After calculating HQ, CR, and RI values for every sampling point in the study area, spatial plots were prepared by applying the natural neighbour interpolation method.

**Figure 4:**
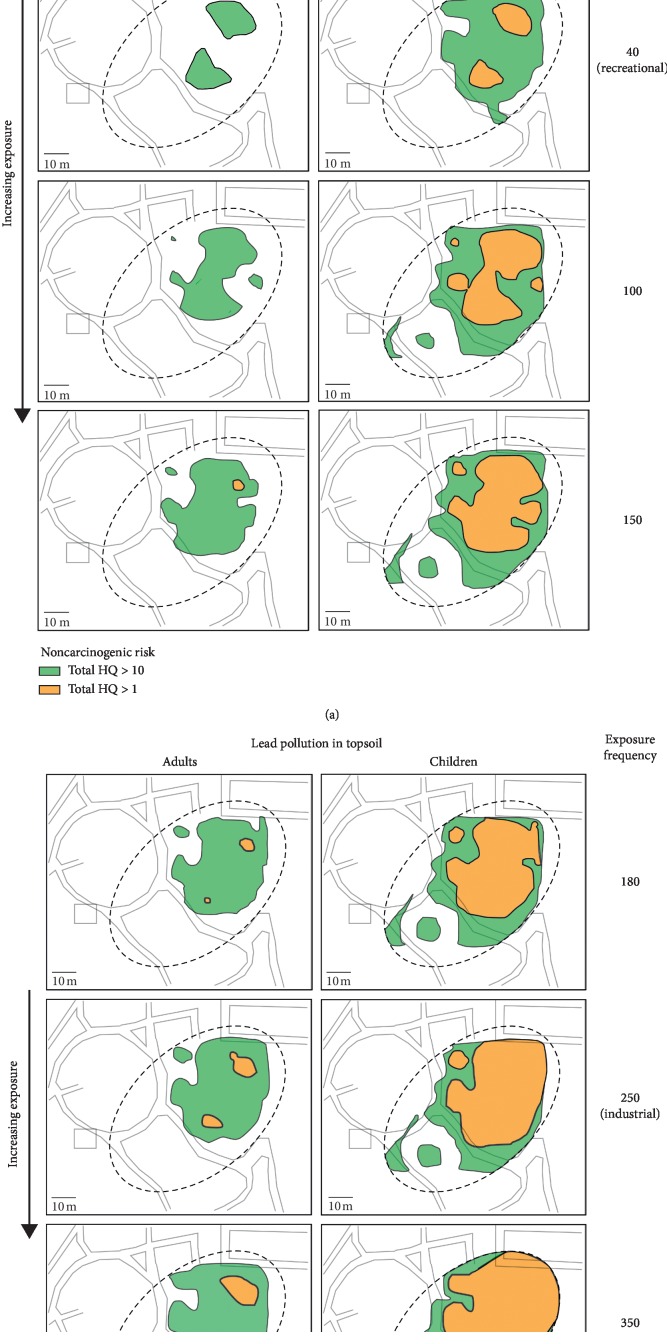
Spatial representation of noncarcinogenic Pb-related risk for adults and children based on different exposure frequencies.

Figure 4 shows a spatial representation of Pb-related noncarcinogenic hazard for adults and children, with different exposure frequency values. It can be seen that the risk area increases with similarly increasing exposure. On an excavation land use scenario, the risk for adults is minimal and the risk for children is mostly present in the top-right corner of the area. On a recreational land use scenario, the risk for adults starts to appear and the risk for children covers more than half of the study area with patches of >10 risk. On an industrial land use scenario, the risk for adults is very noticeable and the risk for children has spread out as well as the >10 risk area. Finally, on a residential land use scenario, the risk for adults covers more than half of the study area with patches of >10 risk, and the risk for children almost covers the whole study area with a big proportion being >10 risk area. It can be noted from the figure that the area in green (HQ > 1) increases in size with increasing exposure, as well as spots of higher risk (area in orange, HQ > 10) appear similarly.

There are very noticeable differences between the plots for Pb-related noncarcinogenic hazard in the study area, and there lies the importance of defining the exposure frequency correctly. Situations where heavy metal concentrations yield risk indicators close to the tolerable limits should include a finer analysis of population exposure to determine the most precise risk value. Similarly, study areas where occupational changes will be made in the future due to varying land use or urbanization evolution should take this information into account to prevent threats to the population and the environment.

**Figure 5:**
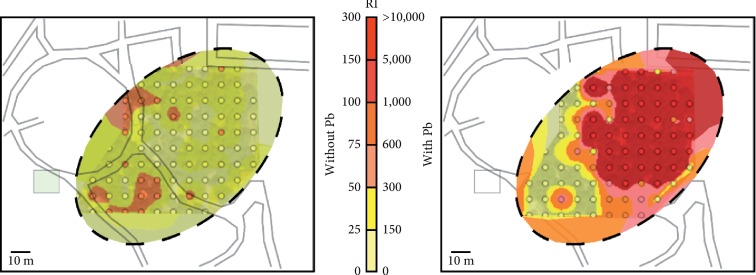
Spatial representation of ecological risk assessment in absence and presence of Pb in the study area.

Regarding ecological risk, Figure 5 shows a comparison of the hypothetical RI spatial distribution excluding and including the presence of Pb in the study area. For this particular site, RI values range from 10 to 84 with a mean of 34 when not considering Pb, which does not result in any risk level. However, when factoring Pb as part of the study area, RI values range from 40 to >10,000 with a mean of 2,022, which results in very high ecological risk. In fact, taking Pb into account, it can be seen that there are only small portions of the area where the ecological risk is <150 suggesting that the majority of the study site suffers from some degree of ecological risk. This public place is an example of the importance of analyzing Pb in topsoil from former shooting ranges since it could comprise risks for both humans and the environment.

## 4. Conclusions

This study has analyzed the influence of Pb exposure in the assessment of human health and ecological risk. High concentrations of Pb were found across a study area. For adults and children, human health risk indicators HQ and CR show the presence of Pb-related noncarcinogenic hazard and a tolerable Pb-related carcinogenic risk, respectively. High ecological risk is also found by using the indicator RI. An analysis of the variations in human health risk indicators due to Pb exposure has led to the conclusion that land use scenarios play an important role in the determination of risks both generally and spatially. Situations where metal concentrations yield human health risk indicators close to tolerable zones should define exposure frequencies precisely. This analysis, in turn, should be granular in scenarios of possible land use changes or areas with future urbanization. Appropriately defining exposure values according to land use will allow local authorities and policy makers to perform and design monitoring and treatment procedures suitably.

## Figures and Tables

**Table 1:** Parameters and values used in human health risk assessment.

Parameter	Name	Units	Value
C_exp_	Concentration of the trace element	ppm	Element dependent
R_ing_	Ingestion rate	mg/day	200, children
			100, adults
*EF*	*Exposure frequency*	*days/year*	*40*, *recreational*
ED	Exposure duration	years	6, children
			24, adults
BW	Body weight	kg	15, children
			70, adults
AT	Averaging time	days	ED × 365
R_inh_	Inhalation rate	m^3^/day	7.5, children
			20, adults
PEF	Particle emission factor	m^3^/kg	1.36 × 10^9^
SA	Exposed skin area	cm^2^/day	2800, children
			5700, adults
SAF	Skin adherence factor	mg/cm^2^	0.2, children
			0.07, adults
ABS	Dermal absorption factor	—	0.001, noncarcinogenic
			0.01, carcinogenic

## Data Availability

The metal concentrations and other data used to support the findings of this study are available from the corresponding author upon request.
